# Cytochrome P450 activity in rheumatoid arthritis patients during continuous IL-6 receptor antagonist therapy

**DOI:** 10.1007/s00228-023-03578-1

**Published:** 2023-10-13

**Authors:** Ann-Cathrine Dalgård Dunvald, Kasper Søltoft, Ekta Sheetal, Søren Andreas Just, Ida Emilie Brejning Frederiksen, Flemming Nielsen, Dorte Aalund Olsen, Jonna Skov Madsen, Oliver Hendricks, Tore Bjerregaard Stage

**Affiliations:** 1 Clinical Pharmacology, Pharmacy and Environmental Medicine, Department of Public Health, University of Southern Denmark, JB Winsløwsvej 19, 2, DK-5000 Odense C, Denmark; 2 Department of Rheumatology, Odense University Hospital, Odense, Denmark; 3 Department of Rheumatology, Hospital South West Jutland, Esbjerg, Denmark; 4 Section of Rheumatology, Department of Medicine, Svendborg Hospital, Odense University Hospital, Svendborg, Denmark; 5 Department of Biochemistry and Immunology, Lillebaelt Hospital, Vejle, Denmark; 6 Department of Regional Health Research, Faculty of Health Sciences, University of Southern Denmark, Odense, Denmark; 7 Danish Hospital for Rheumatic Diseases, Hospital South Jutland, Sønderborg, Denmark; 8 The DANBIO Registry, Glostrup, Denmark; 9 Department of Clinical Pharmacology, Odense University Hospital, Odense, Denmark

**Keywords:** Rheumatoid arthritis, Drug-disease-drug interactions, IL-6 receptor antagonist, Inflammation

## Abstract

**Abstract:**

**Background:**

Inflammation suppresses cytochrome P450 (CYP) enzyme activity, and single-dose interleukin 6 receptor antagonists (anti-IL-6R) reverse this effect. Here, we assess the impact of continuous anti-IL-6R therapy in patients with rheumatoid arthritis.

**Methods:**

In a clinical pharmacokinetic trial, the Basel cocktail was administered before and after 3 and 12 weeks of anti-IL-6R therapy to assess CYP enzyme activity *(registered in the ClinicalTrials.gov database (identifier NCT04842981) on April 13*^*th*^*, 2021)*. In a retrospective study, the 4β-hydroxycholesterol/cholesterol ratio was measured as a biomarker for CYP3A4 activity before and after 3 and 6 months of anti-IL-6R therapy. The control group was patients initiating a tumor necrosis factor alfa (TNF-α) inhibitor.

**Results:**

In the clinical pharmacokinetic trial (*n* = 3), midazolam metabolic ratio (CYP3A4) was inconclusive due to the limited sample size. Midazolam AUC and *C*_max_ indicate a weak impact on CYP3A4 activity after 3 weeks of anti-IL-6R therapy compared to baseline (AUC geometric mean ratio (GMR): 0.80, 95% CI: 0.64–0.99 and *C*_max_ GMR: 0.58, 95% CI: 0.37–0.91), which returns to baseline levels after 12 weeks of therapy (AUC GMR 1.02, 95% CI: 0.72–1.46 and *C*_max_ GMR 1.03, 95% CI 0.72–1.47). No effect on the 4β-hydroxycholesterol/cholesterol ratio was observed in the retrospective study.

**Conclusion:**

Based on sparse data from three patients, continuous anti-IL-6R therapy seems to cause an acute but transient increase in CYP3A4 activity in rheumatoid arthritis patients, which may be due to a normalization of the inflammation-suppressed CYP activity. Further studies are warranted to understand the mechanism behind this putative transient effect.

*Trial registration *Registered in the ClinicalTrials.gov database (identifier NCT04842981) on April 13th, 2021.

**Supplementary Information:**

The online version contains supplementary material available at 10.1007/s00228-023-03578-1.

## Introduction

**Fig. 1:**
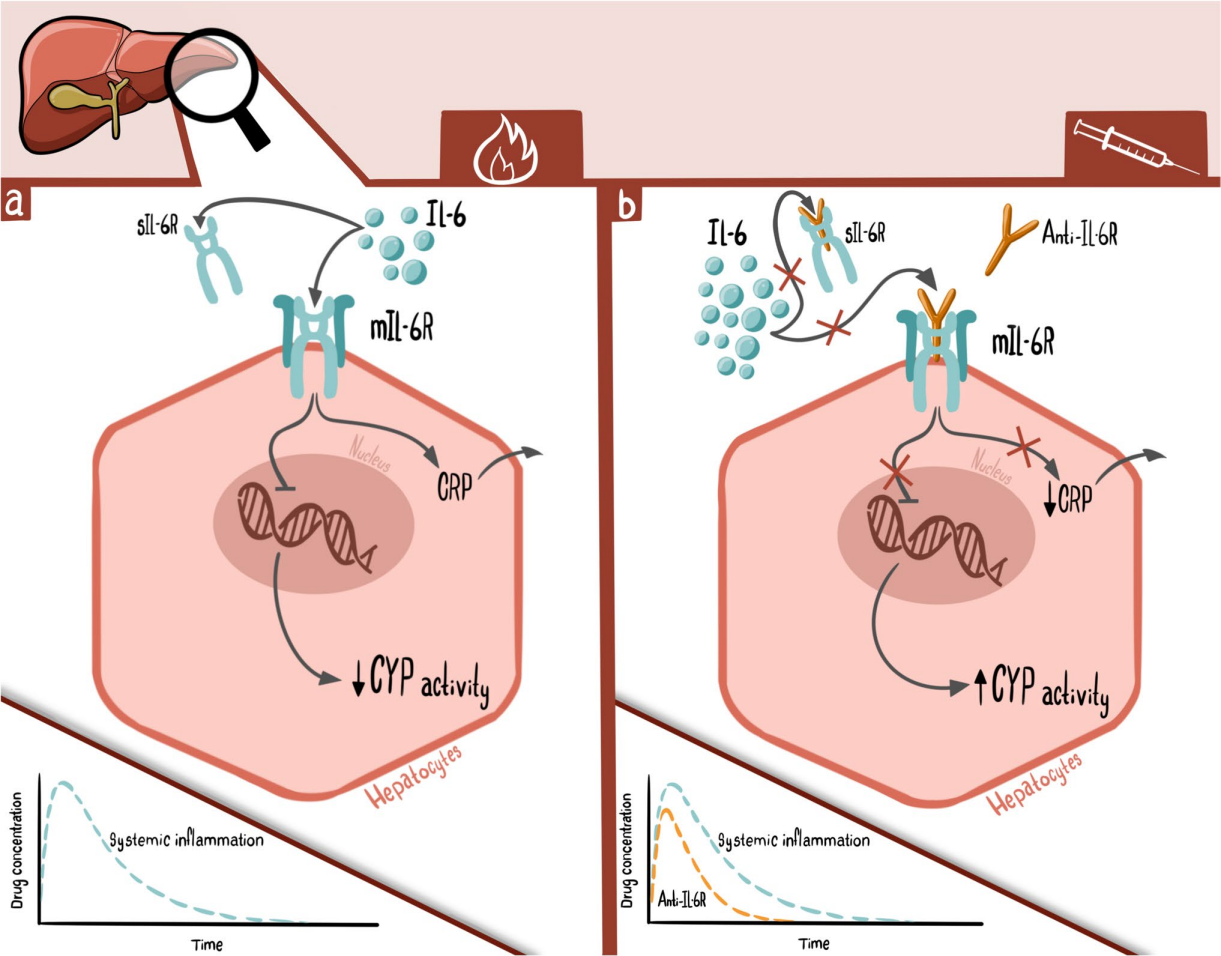
Interleukin (IL)-6 is crucial to rheumatoid arthritis pathophysiology. **a** IL-6 binds to the soluble IL-6 receptor (sIL-6R) and membrane-bound IL-6 receptor (mIL-6R) and affects numerous intracellular pathways. In the hepatocytes, IL-6 is a significant contributor to the downregulation of the transcription of cytochrome p450 (CYP) enzymes, leading to lower drug-metabolizing activity and increased drug concentrations. The pathways leading to altered CYP transcription are not entirely elucidated to date. **b** IL-6 receptor antagonists (anti-IL-6R) are used to treat rheumatoid arthritis. Anti-IL-6R blocks the receptors and disrupts the intracellular pathway otherwise activated by inflammation. Blockade of IL-6 causes a normalization of drug-metabolizing capacity and a reduction in drug concentrations. Anti-IL-6R therapy increases the IL-6 concentration, while c-reactive protein is reduced. Abbreviations: anti-IL-6R, interleukin 6 receptor antagonist; CRP, c-reactive protein; CYP, cytochrome P450; IL-6, interleukin 6; mIL-6R, membrane-bound interleukin 6 receptor; sIL-6R, soluble interleukin 6 receptor

Rheumatoid arthritis is a chronic autoimmune disease characterized by systemic and local joint inflammation. The adaptive and innate immune systems are activated and involved in the inflammatory response, and interleukin (IL) 6 plays a crucial role in the pathogenesis of rheumatoid arthritis [1]. Besides the local inflammatory effects, systemic IL-6 impacts the disease burden in patients with rheumatoid arthritis [1]. In the liver, IL-6 upregulates the acute-phase protein c-reactive protein (CRP) (Fig. 1) [2]. Moreover, IL-6 is involved in the regulation of drug-metabolizing cytochrome P450 (CYP) enzymes (Fig. 1) [3–5].

In 2009, the first humanized IL-6 receptor antagonist (anti-IL-6R) monoclonal antibody, tocilizumab, was approved by the European Medicines Agency for the treatment of rheumatoid arthritis [6]. Since, the equivalent anti-IL-6R sarilumab has also been approved for rheumatoid arthritis [7]. Both therapies effectively relieve pain symptoms and prevent further synovial destruction caused by inflammation in rheumatoid arthritis [6, 7]. The mechanism of action is selective binding to the soluble and membrane-bound IL-6 receptor (sIL-6R and mIL-6R), thus inhibiting the binding of IL-6 and the subsequent signaling cascades (Fig. 1) [6, 7]. The formation of drug-receptor complexes depends on free anti-IL-6R drug concentrations [2]. Following treatment with an anti-IL-6R, the concentration of sIL-6R rapidly increases within 2 weeks and reaches a steady state after 12–16 weeks [3, 8, 9]. Accordingly, a significant reduction in CRP is observed within 2 weeks of the first anti-IL-6R dose [3, 8, 9].

High concentrations of IL-6 suppress the expression of drug-metabolizing enzymes [5]. Anti-IL-6R potentially reverses this suppression, causing upregulation of CYP enzymes and possibly leading to drug-disease-drug interactions with other drugs of potential clinical importance [10]. In vitro, anti-IL-6R is reported to normalize the otherwise IL-6-suppressed expression of CYP1A2, CYP2C9, CYP2C19, and CYP3A4 [6, 7, 11]. Clinical studies have shown a > 50% lower simvastatin (CYP3A4 substrate) exposure 1 week after a single anti-IL-6R dose in patients with rheumatoid arthritis [12, 13], indicating a normalization (increase) in CYP3A4 activity. To date, no studies have assessed the interaction potential following multiple doses of anti-IL-6R, thus reflecting a realistic course of treatment. We hypothesized that the impact on CYP enzymes by anti-IL-6R becomes more pronounced at the steady state of anti-IL-6R therapy.

Specific phenotyping probe drugs are often utilized to assess drug-drug interactions, sometimes in combination as phenotyping cocktails [14]. The biomarker 4β-hydroxycholesterol has been proposed as an endogenous biomarker of CYP3A4 activity [15]. 4β-Hydroxycholesterol is a cholesterol metabolite formed via CYP3A4 and changes in the concentration of 4β-hydroxycholesterol or the 4β-hydroxycholesterol/cholesterol ratio might reflect changes in CYP3A4 mediated metabolism [16]. 4β-Hydroxycholesterol has an elimination half-life of 17 days, seemingly allowing the assessment of slow and long-term changes in CYP3A4 activity [15].

This study aimed to prospectively assess the activity of six CYP enzymes in patients with rheumatoid arthritis before and after 3 and 12 weeks of continuous anti-IL-6R therapy. Furthermore, to translate these findings into clinical impact, we aimed to assess this potential drug-disease-drug interaction utilizing the biomarker 4β-hydroxycholesterol in a retrospective study of incident tocilizumab users.

## Method

We conducted two studies: [1] a prospective clinical pharmacokinetic trial including patients with rheumatoid arthritis and indication for initiation of anti-IL-6R treatment and [2] a retrospective study of patients with rheumatoid arthritis who provided samples for the Danish Rheumatologic Biobank.

### Design and setting

#### Clinical pharmacokinetic trial

We conducted a clinical pharmacokinetic trial to assess the impact of initiating anti-IL-6R on the activity of drug-metabolizing enzymes in patients with rheumatoid arthritis. From May 2021 to September 2022, we included patients with active rheumatoid arthritis and an indication for treatment with an anti-IL-6R. Potential trial patients were pre-screened, and those who fulfilled the in- and exclusion criteria were asked to participate. Most criteria were based on restrictions and warnings in the summary of product characteristics (SmPC). The patients should be 18–75 years old and have active rheumatoid arthritis. Estimated glomerular filtration rate (eGFR), absolute neutrophil count (ANC), platelet count, and alanine transaminase (ALAT) should be normal or < 3 × upper limit of normal, in concordance with the SmPC. Test for hepatitis and tuberculosis should be negative. Patients with severe infections, diverticulitis, or malignancies were excluded. Intake of medication or herbal medicines potentially interacting with the administered probe drugs was prohibited. Concurrent conventional disease-modifying antirheumatic drugs (DMARDs) and concomitant glucocorticoid therapy were allo°wed at the discretion of the treating rheumatologist.

In this trial, subcutaneous (s.c.) tocilizumab or sarilumab were eligible interventions, and the therapy of choice was individualized. Patients receiving tocilizumab self-administered 162 mg s.c. every week, and patients receiving sarilumab self-administered 200 mg s.c. every other week. Patients recorded the time of administration to ensure compliance. Clinical data were obtained from medical records, and follow-up was concluded 3 months after the last trial day (12 weeks).

The trial was designed as a self-controlled trial, and each patient acted as their own control. The initiation of an anti-IL-6R was defined as day 0. The patients attended three trial days: before initiation of the anti-IL-6R therapy (baseline; day − 7 ± 5 days), after 3 weeks of anti-IL-6R therapy (day 21 ± 10 days), and after 12 weeks of anti-IL-6R therapy (day 84 ± 10 days). Patients were restricted from consuming bitter orange, grapefruit, alcohol, caffeine, and theobromine 48 h before each trial day. Following an overnight fast, patients ingested the Basel cocktail [17, 18], consisting of 100 mg caffeine (Cofi-Tabs (dietary supplement), Vitabalans, Finland), 50 mg efavirenz (Stocrin^®^, MSD, Denmark), 12.5 mg losartan (Losartan “Medical Valley,” Medical Valley, Sweden), 10 mg omeprazole (Omeprazole “Medical Valley,” Medical Valley, Sweden), 12.5 mg metoprolol (Metoprololsuccinat “Hexal,” Sandoz, Denmark), and 2 mg midazolam (oral solution, Ozalin, Primex Pharmaceuticals, Finland). Blood samples were drawn through an intravenous catheter at times 0 (before administration of the Basel cocktail), 0.25, 0.5, 0.75, 1, 2, 3, 4, and 6 h. Blood was collected in serum-separating tubes; samples for plasma were collected in K2-EDTA tubes and centrifuged at 2500 g for 10 min. Samples for serum were collected in serum-separating tubes and were coagulated for 30 min, after which they were centrifuged at 2000 g for 10 min. Urine was collected at 0–6 h. Plasma, serum, and urine were transferred to cryo-tubes and stored at − 20 °C for in-house analysis and − 80 °C for long-term storage of samples to be analyzed externally.

#### Retrospective study

We conducted a retrospective self-controlled study to assess the activity of CYP3A4 measured by the biomarker 4β-hydroxycholesterol in patients with rheumatoid arthritis before and after initiating the anti-IL-6R tocilizumab. Patients with rheumatologic diseases treated at Danish hospitals or private rheumatologic clinics are registered in DANBIO [19]. Information on patient demographics, treatment, patient-reported and clinical outcomes, and comorbidity and lifestyle factors are registered in DANBIO. Within DANBIO, we identified patients with rheumatoid arthritis at the Danish Hospital for Rheumatic Diseases, Sønderborg. In the tocilizumab group, we included patients registered to initiate tocilizumab in the period between 2016 and 2021. As controls, we identified a cohort of patients with rheumatoid arthritis initiating the tumor necrosis factor alfa (TNF-α) inhibitor adalimumab in the same period. Biological samples (serum) from patients in the tocilizumab and adalimumab group were retrieved at time points immediately before initiation of treatment and after 3 and 6 months of treatment according to biobank protocol. At least one sample was required during follow-up (3 or 6 months). Samples were obtained from the Danish Rheumatologic Biobank [20] hosted by the Bio- and Genome Bank Denmark. In the biobank, samples are collected prospectively from patients with rheumatic disease before and during treatment [21].

### Patient research partners

Patients from the user council of the Danish Hospital for Rheumatic Diseases, Sønderborg, have been involved in the conception of the study. The patients and their perspectives have been a part of developing the patient information brochures.

### Analytical methods

All analyses were conducted after the studies and were blinded from clinical data.

In the clinical pharmacokinetic trial, drugs and metabolites were analyzed in plasma and urine at the Department of Public Health, University of Southern Denmark. We quantified caffeine, paraxanthine, efavirenz, 8-hydroxyefavirenz, losartan, E3174, metoprolol, hydroxymetoprolol, midazolam, and α-hydroxymidazolam using high-performance liquid chromatography (LC) and high-resolution mass spectrometry (HR-MS). The method is previously described in detail [22] and follows a previously utilized approach with minor modifications [17, 23–25]. We used quality control (QC) samples, blanks, and calibration curves in the batch to assess precision and accuracy. The within-day and between-day precision and accuracy were < 15% for all compounds. The lower limit of quantification (LLOQ) and upper limit of quantification (ULOQ) ranged from 0.5 to 5 ng/mL to 500 to 2000 ng/mL, respectively, for the individual compounds.

In the clinical pharmacokinetic trial, patients were genotyped to identify the most common *CYP2C9* alleles (*1, *2, *3) and *CYP2D6* alleles (*1, *2, *3, *4, *5, *6, *9, *10, *17, and *41 or gene duplicates). We used a previously validated 5’ nuclease real-time polymerase chain reaction (PCR) panel [26].

Samples from the clinical pharmacokinetic trial and the retrospective study were analyzed for cholesterol and 4β-hydroxycholesterol concentrations at the Department of Public Health, University of Southern Denmark. The concentration of 4β-hydroxycholesterol was determined using a previously described method [27]. Detection was performed with a triple quadrupole mass spectrometer (MS/MS). The analytical system consisted of a Dionex Ultimate 3000 RS UHPLC pump, a CTC autosampler, and a TSQ Quantiva mass spectrometer. The mass spectrometer was operated in positive polarity with Atmospheric Pressure Chemical Ionization (APCI). The ion discharge current was 5 μA, the sheath gas was 45 arbitrary units (AU), the auxiliary gas was 5 AU, the sweep gas was 1 AU, the ion transfer tube temperature was 325 °C, and the vaporizer temperature was 350 °C. The ion transitions for monitoring 4β-hydroxycholesterol were *m/z* 385.4 > 109.2 as quantifier trace (collision energy 27 V) and *m/z* 385.4 > 367.4 as qualifier trace (collision energy 14 V). d7-4β-Hydroxycholesterol (provided from Avanti Polar Lipids Inc.; Alabaster, AL, USA) was used as an internal standard and monitored at the transition *m/z* 392.2 > 109.1 (collision energy 27 V). The within- and between-batch variation (CV%) of the QC samples was less than 13%. LLOQ was 5 ng/mL, and ULOQ was 1000 ng/mL.

The cholesterol concentration in plasma was determined using a Thermo Scientific cholesterol kit on a Konelab 20 Clinical Chemistry Autoanalyzer (Thermo Scientific, Vantaa, Finland). Calibrators, blanks, and QC samples for normal values and abnormal (high) levels (Nortrol and Abtrol) were included in the batch of samples analyzed. The within- and between-batch variation (CV%) of the QC samples was less than 1%. The precision determined as bias was − 2% for both types of QC samples. LLOQ was 0.1 mmol/L (40 μg/mL) and ULOQ was 15 mmol/L (5770 μg/mL).

The inflammatory markers were analyzed at the Department of Biochemistry and Immunology, Lillebælt Hospital, using Roche/Hitachi cobas (hsCRP and IL-6) or Simoa HD-X analyzer (IL-1β, TNF-α, and IFN-γ) (Quanterix^©^, Billerica, MA, USA). Assay quality control was performed using two controls prepared from commercially available control material provided by the manufacturer and in-house prepared serum pools for the Simoa assays. The analytical coefficients of variation were calculated to be < 16% in the IL-1β assay, < 13% in the TNF-α assay, and < 6% in the INF-γ. The within-run and between-run precision were < 8.6% for the hsCRP and IL-6 assays. According to the kit insert, hsCRP has a LOD of 0.15 mg/L and a LOQ of 0.3 mg/L. The remaining inflammatory markers have LOD of 0.015 to 1.5 pg/mL and LOQ of 0.034 to 2.5 pg/mL.

Samples from all patients treated with tocilizumab in the clinical pharmacokinetic trial and the retrospective study were included in commercially available immunoassays to quantify tocilizumab (Aybaytech, Yenimahalle, Turkey), IL-6 receptor (R&D Systems, Minneapolis, MN, USA), and antibodies to tocilizumab (Aybaytech). The manufacturer’s procedures were followed. In-house prepared quality controls were included in each run to evaluate assay performances. The samples and the assay controls were analyzed in duplicates, and the mean concentration was reported. The analytical coefficients of variation were 8–13%.

### Sample size

In the prospective study, the primary endpoint was a difference in the metabolic ratio of midazolam at 3 weeks of anti-IL-6R treatment compared to baseline. We aimed to include 12 patients in the study to detect a ≥ 35% difference in midazolam metabolic ratio with 80% power, a two-sided significance level of 5%, and a drop-out rate of 20%. The COVID-19 pandemic significantly delayed recruitment, and the trial was prematurely terminated on September 30th, 2022. In the retrospective study, no sample size calculation was conducted; thus, all available samples in the specified period were included in the analysis. Statistically or clinically relevant differences were not defined prior to study conduct.

### Statistics and pharmacokinetic analysis

Demographic data and pharmacokinetic parameters are presented as medians and interquartile ranges (IQR; 25th to 75th percentiles) or geometric mean ratios (GMR) with a 95% confidence interval. Non-compartmental analysis was conducted as previously described [28] using the R package PKNCA [29]. The metabolic ratio was calculated as [drug]/[metabolite] at the time points previously validated to correspond to the AUC ratio [17]. The formation clearances (CL_f_) were estimated as [amount of metabolite in urine_0–6 h_] over [AUC of substate_0–6 h_], and renal clearances (CL_R_) were calculated as [amount of substrate in urine_0–6 h_] over [AUC of substate_0–6 h_].

## Results

### Clinical pharmacokinetic trial

The trial was prematurely concluded and did not obtain the pre-defined sample size. We included three patients with rheumatoid arthritis from three trial sites. Patients were between 54 and 72 years old, two were female, and none were smokers. Disease duration was registered for two patients and ranged from 13 to 18 years. All patients were previously treated with other biological DMARDs. Two patients received concomitant treatment with a conventional DMARD during the trial. All patients initiated an anti-IL-6R; two received subcutaneous sarilumab, and one received tocilizumab. The 3- and 12-week phenotyping was conducted on days 16 to 22 and 81 to 86 after initiation of treatment, respectively. No adverse events related to the cocktail drugs were reported.

**Fig. 2:**
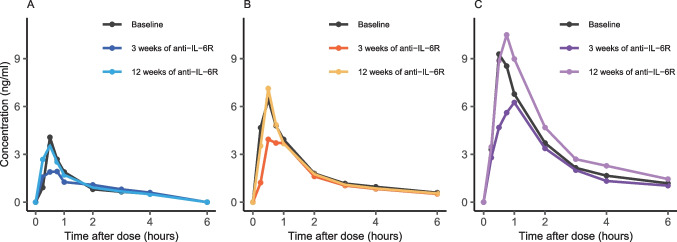
Individual concentration–time curves of midazolam for the three patients with rheumatoid arthritis before and after 3 weeks and 12 weeks of interleukin 6 receptor antagonist (anti-IL-6R) therapy

Midazolam metabolic ratio showed inconclusive results after 3 and 12 weeks of anti-IL-6R therapy compared to baseline, due to the limited sample size; at baseline, the median midazolam metabolic ratio ([mid]/[OH-mid]) was 0.12 (IQR: 0.09–0.13) and was slightly decreased after 3 weeks to a median 0.08 (IQR: 0.07–0.09; GMR: 0.82, 95% CI: 0.47–1.44). At 12 weeks, [mid]/[OH-mid] once again approached baseline levels (median 0.15 (IQR: 0.11–0.15); GMR: 1.16, 95% CI: 0.86–1.57). In line, the individual pharmacokinetic metrics indicated a weak increase in CYP3A4 activity after 3 weeks of anti-IL-6R therapy. Midazolam area under the plasma concentration curve (AUC) from 0 to ∞ decreased by 20% (GMR: 0.80, 95% CI: 0.64–0.99), and maximum plasma concentration (*C*_max_) decreased by 42% (GMR: 0.58, 95% CI: 0.37–0.91) (Fig. 2). Following 12 weeks of repeated anti-IL-6R therapy, the effect seems diminished, and both AUC_0–∞_ and *C*_max_ returned to the baseline level (AUC_0–∞_ GMR: 1.02, 95% CI: 0.72–1.46 and *C*_max_ GMR: 1.03, 95% CI: 0.72–1.47). This observation was consistent for all three trial subjects (Fig. 2 and Table S1). For caffeine (CYP1A2), efavirenz (CYP2B6), losartan (CYP2C9), and omeprazole (CYP2C19), the interindividual variation is too large to observe any patterns based on the small sample size (Table S1 and Fig. S1). One individual was genotyped as a CYP2C19 ultrarapid metabolizer (CYP2C19*1/*17). Metoprolol (CYP2D6) was excluded from the analysis due to concentrations below the limit of quantification of the drug or metabolite. 4β-Hydroxycholesterol and 4β-hydroxycholesterol/cholesterol ratio were unchanged following 3 or 12 weeks of treatment with an anti-IL-6R among the three patients (Table S2).

All three patients had low levels of inflammation at baseline (Table 1). hsCRP showed a marked reduction after 3 weeks of anti-IL-6R therapy, which remained at 12 weeks of treatment. In line, IL-6 increased after 3 weeks and 12 weeks of anti-IL-6R therapy. IL-1β, TNF-α, and IFN-γ remained at the same level at baseline and after 3 and 12 weeks of anti-IL-6R therapy (Table 1).

**Table 1:** Initiation of IL-6 receptor antagonist therapy in three patients with rheumatoid arthritis causes a quick (3 weeks) and significant (12 weeks) decrease in hsCRP and an increase in IL-6

Parameter	Baseline	3 weeks	3 weeks/baseline	12 weeks	12 weeks/baseline
	Median (IQR)	Median (IQR)	GMR (95% CI)	Median (IQR)	GMR (95% CI)
hsCRP (mg/L)	5.2 (3.2–5.4)	0.6 (0.4–2.1)	0.22 (0.02–2.30)	0.3 (0.2–0.3)	0.08 (0.02–0.36)
IL-6 (pg/mL)	4.1 (3.4–4.8)	92.2 (55.5–104.1)	15.06 (1.06–214.22)	67.0 (47.0–67.6)	12.81 (2.35–69.89)
IL-1β (pg/mL)	0.1 (0.1–0.1)	0.1 (0.1–0.1)	0.83 (0.74–0.92)	0.1 (0.1–0.1)	0.83 (0.27–2.62)
TNF-α (pg/mL)	2.6 (2.5–4.5)	3.0 (2.8–3.8)	0.97 (0.48–1.95)	3.8 (3.1–4.6)	1.09 (0.48–2.48)
IFN-γ (pg/mL)	0.6 (0.4–1.6)	0.6 (0.4–1.5)	0.84 (0.43–1.64)	1.5 (0.8–5.3)	2.07 (0.41–10.60)

*CI* confidence interval, *GMR* geometric mean ratio, *hsCRP* high-sensitivity c-reactive protein, *IFN* interferon, *IL* interleukin, *IQR* interquartile range, *TNF* tumor necrosis factor

### Retrospective study

In the retrospective study, we included a tocilizumab group with 20 patients and an adalimumab group with 17 patients as control. Demographic data are presented in Table 2. The groups were similar in sex and age. However, the groups differed by previous and current antirheumatic treatment. In line with the recommendations [30], half of the patients in the tocilizumab group received concurrent therapy with a conventional DMARD, and > 80% of patients in the adalimumab group received concurrent treatment with a conventional DMARD. Almost 95% of the patients in the tocilizumab group had previously been treated with one or more biological medicines according to clinical guidelines [31, 32]. In contrast, only 44% in the adalimumab group had previously received one or more biological treatments (Table 2).

**Table 2:** Demographic characteristics of the two groups at baseline, prior to initiation of tocilizumab and adalimumab, respectively

	**Tocilizumab**	**Adalimumab**
Total, *n*	20	17
Female sex, *n* (%)	14 (70%)	13 (76%)
Age, median (IQR)	62 (61–72)	61 (49–71)
Time since diagnosis (years), median (IQR)^*^	12 (5–18)	10 (2–18)
RA diagnosis^*^		
Seronegative M06.0	1 (6%)	5 (31%)
Seropositive M05.9	16 (89%)	10 (63%)
Unspecified M06.9	1 (6%)	1 (6%)
Current DMARDS, ***n*** (%)^*^	9 (50%)	13 (81%)
Methotrexate	1 (11%)	9 (69%)
Salazopyrin	5 (56%)	9 (69%)
Leflun	2 (22%)	0 (0%)
Chloroquine	1 (11%)	6 (46%)
Current prednisolon, ***n*** (%)^*^	7 (39%)	4 (25%)
Prior biologics, *n* (%)^*^	17 (94%)	7 (44%)
Abatacept	2 (12%)	3 (43%)
Adalimumab	7 (41%)	-
Certolizumab	3 (18%)	1 (14%)
Etanercept	11 (65%)	2 (29%)
Golimumab	1 (6%)	-
Infliximab	3 (18%)	3 (43%)
Rituximab	-	1 (14%)
Tofacitinib	4 (24%)	1 (14%)

^*^Data available for *n* = 18 in the tocilizumab group and *n* = 16 in the adalimumab group

In the tocilizumab and adalimumab groups, the 4β-hydroxycholesterol/cholesterol ratio remained stable after 3 and 6 months of treatment (Table 3), indicating no clinically meaningful change in CYP3A4-mediated metabolism. In the tocilizumab group, 4β-hydroxycholesterol increased following 3 months of anti-IL-6R therapy (GMR 1.13, 95% CI 1.02–1.25), accompanied by a continuous increase in cholesterol at 3 and 6 months (Table 3). The 4β-hydroxycholesterol decreased slightly towards baseline after six months of anti-IL-6R therapy (6 months/baseline GMR 1.04, 95% CI 0.89-1.22). No changes in 4β-hydroxycholesterol, cholesterol, or 4β-hydroxycholesterol/cholesterol ratio were observed in the adalimumab group (Table 3).

**Table 3:** 4β-Hydroxycholesterol/cholesterol ratio as a biomarker of CYP3A4 activity was unchanged after 3 and 6 months of therapy in patients treated with tocilizumab or adalimumab

Parameter	Baseline Median (IQR)	3 months Median (IQR)	3 months/baseline GMR (95% CI)	6 months Median (IQR)	6 months/baseline GMR (95% CI)
Tocilizumab					
Cholesterol µg/mL	2000 (1549–2340)	2111 (1896–2354)	1.10 (1.04–1.17)	2224 (1947–2649)	1.14 (1.05–1.23)
4β-OHC ng/mL	43.3 (33.3–73.8)	52.9 (36.6–77.1)	1.13 (1.02–1.25)	51.6 (33.8–84.5)	1.04 (0.89–1.22)
4β-OHC/cholesterol^a^	0.21 (0.18–0.33)	0.24 (0.19–0.33)	1.03 (0.94–1.12)	0.23 (0.16–0.35)	0.91 (0.81–1.03)
Adalimumab					
Cholesterol µg/mL	1875 (1835–2294)	2001 (1830–2144)	0.99 (0.88–1.12)	2074 (1777–2269)	1.02 (0.9–1.15)
4β-OHC ng/Ml	43.6 (29.7–54.3)	43.6 (30.2–59.0)	1.02 (0.87–1.21)	35.4 (26.1–51.4)	0.93 (0.75–1.15)
4β-OHC/cholesterol^a^	0.19 (0.13–0.28)	0.21 (0.14–0.30)	1.03 (0.90–1.18)	0.16 (0.11–0.26)	0.91 (0.76–1.09)

*4*β*-OHC* 4 beta-hydroxycholesterol, *CI* confidence interval, *IQR* interquartile range, *GMR* geometric mean ratio

^a^Ratio denoted as 4β-OHC (ng/mL)/cholesterol (ng/mL) ^*^10^4^

**Fig. 3:**
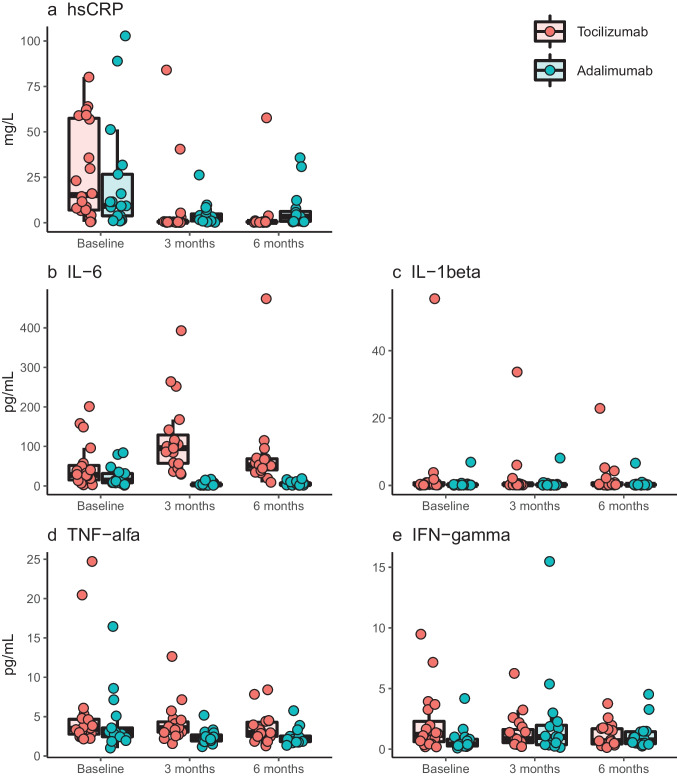
Initiation of treatment with tocilizumab (*n* = 20) or adalimumab (*n* = 17) is associated with significant changes in inflammation after 3 months and 6 months of treatment. **a** High-sensitivity c-reactive protein (hsCRP) (mg/L). **b** Interleukin 6 (IL-6) (pg/mL). **c** Interleukin 1beta (IL-1β) (pg/mL). **d** Tumor necrosis factor alpha (TNF-α) (pg/mL). **e** Interferon-gamma (IFN-γ) (pg/mL)

Initiation of treatment was associated with statistically significant changes in inflammation after 3 and 6 months of treatment in the tocilizumab and the adalimumab groups (Fig. 3). In the tocilizumab group, hsCRP was suppressed at 3 months and 6 months, while IL-6 was higher at both follow-ups compared to baseline. TNF-α was lower following 6 months of tocilizumab therapy compared to baseline. In the adalimumab group, hsCRP, IL-6, and TNF-α were lower after 3 and 6 months of treatment. In contrast, IFN-γ was higher after 3 and 6 months of adalimumab therapy compared to baseline (Table 4 and Fig. 3). In the tocilizumab group, the soluble IL-6 receptor concentrations increased during anti-IL-6R therapy (Table 4).

**Table 4:** Therapy with tocilizumab (*n* = 20) or adalimumab (*n* = 17) causes significant alterations to the ongoing inflammation in patients with rheumatoid arthritis following 3 months and 12 months of treatment compared to baseline

Parameter	Baseline Median (IQR)	3 months Median (IQR)	3 months/baseline GMR (95% CI)	6 months Median (IQR)	6 months/baseline GMR (95% CI)
**Tocilizumab**					
hsCRP (mg/L)	15.2 (7.0–57.5)	0.5 (0.3–0.7)^a^	0.06 (0.03–0.12)	0.5 (0.3–0.6)^b^	0.05 (0.02–0.1)
IL-6 (pg/mL)	27.6 (15.0–51.8)	95.6 (57.2–129.0)^a^	3.52 (2.04–6.06)	52.1 (40.4–68.7)^b^	1.88 (1.12–3.16)
IL-1β (pg/mL)	0.3 (0.1–0.8)	0.3 (0.1–0.5)^a^	0.99 (0.77–1.28)	0.3 (0.2–0.8)^b^	1.03 (0.47–2.27)
TNF-α (pg/mL)	3.5 (2.8–4.7)	3.5 (3–4.3)^a^	0.91 (0.75–1.09)	2.9 (2.5–4.3)^b^	0.76 (0.6–0.96)
IFN-γ (pg/mL)	1.2 (0.6–2.3)	0.8 (0.5–1.6)^a^	0.85 (0.57–1.28)	0.6 (0.4–1.7)^b^	0.74 (0.38–1.46)
IL-6 receptor ng/mL	51.5 (43.5–62.5)	553.8 (452.8–659.0)	7.68 (4.82–12.25)	565.7 (517.8–744.7)	9.38 (6.18–14.23)
Tocilizumab µg/mL	0 (0–0)	26.8 (15.8–59.8)	-	37.3 (25.7–57.7)	-
Tocilizumab antibody ng/mL	0 (0–7.3)	5.2 (0.0–10.7)	-	5.3 (0.0–9.6)	-
**Adalimumab**					
hsCRP (mg/L)	9.3 (3.8–26.6)	2.3 (1.0–4.8)^c^	0.25 (0.13–0.47)	3.3 (0.7–6.2)^c^	0.3 (0.14–0.64)
IL-6 (pg/mL)	13.2 (7.9–31.3)	3.6 (1.7–4.4)^c^	0.22 (0.11–0.44)	4.7 (2.2–7.0)^c^	0.31 (0.16–0.61)
IL-1β (pg/mL)	0.1 (0.1–0.3)	0.1 (0.1–0.3)^c^	0.97 (0.67–1.42)	0.2 (0.1–0.3)^c^	1.05 (0.81–1.35)
TNF-α (pg/mL)	3.0 (2.4–3.6)	2.4 (1.9–2.7)^c^	0.69 (0.52–0.93)	2.1 (1.8–2.5)^c^	0.71 (0.51–0.99)
IFN-γ (pg/mL)	0.4 (0.3–0.8)	1.0 (0.4–2)^c^	2.06 (1.15–3.68)	0.7 (0.4–1.4)^c^	1.64 (1.03–2.6)

*CI* confidence interval, *GMR* geometric mean ratio, *hsCRP* high-sensitivity c-reactive protein, *IFN* interferon, *IL* interleukin, *IQR* interquartile range *TNF* tumor necrosis factor

^a^*n* = 19

^b^*n* = 18

^c^*n* = 16

## Discussion

In this study, we combined a clinical pharmacokinetic trial and a retrospective observational study to assess the impact of continuous anti-IL-6R therapy on CYP-mediated drug metabolism. Based on the primary endpoint midazolam metabolic ratio, we observe a slight trend towards a weak increase in CYP3A4 activity in patients with rheumatoid arthritis after 3 weeks of anti-IL-6R therapy. CYP3A4 activity seems to return to baseline levels after 12 weeks of treatment. However, the limited number of trial subjects restricts any clear conclusions, and further studies are needed to elucidate this potentially transient effect on CYP3A4 activity. We observed no clinically meaningful changes in 4β-hydroxycholesterol/cholesterol ratio.

Inflammation and IL-6 have been linked to decreased CYP enzyme activity, and anti-IL-6R therapy reverses the suppression of CYP enzymes, hence normalizing CYP enzyme activity [12, 13]. Our results indicate that this normalization of CYP activity appears to be temporary and that it returns to baseline during prolonged treatment. Previous studies have shown that CYP activity increases 1 week after a single dose of anti-IL-6R [12, 13]. The activity remains increased 5 weeks after a single dose, though less pronounced [12]. The return of CYP enzyme activity towards the baseline level has previously been interpreted to be a consequence of the diminishing effect and clearance of the single dose of anti-IL-6R. However, our data indicate that CYP enzyme activity will return to baseline following 12 weeks of continuous IL-6R therapy despite IL-6 levels remaining high throughout the study period. While some uncertainty remains on the effect size, due to the limited sample size, the change in CYP3A4 activity seems to be less than twofold, corresponding to a weak interaction. As such, this interaction will likely only be clinically relevant for concomitant drugs with a narrow therapeutic index.

At baseline, the IL-6 levels (median 4.1 pg/mL) in the three patients were below what has previously been described in patients with rheumatoid arthritis (~ 50 pg/mL) [12, 13]. It is unclear if baseline inflammation levels play a part in the impact on CYP3A4 and if this lower grade of inflammation might bias the results and impact the observed transient effect. No apparent effect was observed in the retrospective study assessing the 4β-hydroxycholesterol/cholesterol ratio, possibly due to the prolonged elimination half-life of 4β-hydroxycholesterol. As the observed increase in CYP metabolism is transient, the effect cannot substantially affect β-hydroxycholesterol concentrations, as the altered CYP activity persists for a shorter period than the half-life of 4β-hydroxycholesterol. Overall, these observations could indicate a complex, interconnected regulation of CYP enzymes by cytokines such as IL-6. It might be hypothesized that a fine-tuned negative feedback system mutually regulates the cytokine receptors in hepatocytes, regulating CYP expression and activity. No studies have previously shown that the increase or normalization in CYP3A4 activity caused by anti-IL-6R returns to baseline following continuous treatment. Further studies are warranted to confirm this preliminary finding and elucidate the underlying mechanism.

The main limitation of the clinical pharmacokinetic trial is the limited sample size of only three patients with rheumatoid arthritis, and the obtained results may only be considered preliminary. Anti-IL-6R therapies are not the primary choice of therapy in Denmark, and the enrollment of trial subjects was challenged by their high disease burden at the time of screening, hindering their willingness to participate in the trial. As such, the trial failed to reach the sample size, and more extensive studies are warranted. While we observed an effect on CYP3A4 activity assessed by midazolam pharmacokinetics among the three patients in the clinical trial, this was not supported by the assessment of 4β-hydroxycholesterol in the clinical pharmacokinetic trial. Furthermore, we could not assess the CYP2D6 metabolism due to low concentrations of metoprolol and the metabolite OH-metoprolol. However, previous in vitro studies have not shown an effect on CYP2D6 activity [6, 7, 11]. The trial also has several strengths. First, the long duration of the trial with continuous therapy mimics the treatment course in patients with rheumatoid arthritis. Second, we included rheumatoid arthritis patients with a clinical indication for anti-IL-6R therapy; thus, the results are generalizable to the intended population. Third, we sampled for 6 h following the administration of the Basel cocktail to increase the feasibility for the patient. Last, the main strength of this study is the translational approach, which combines prospective and retrospective observational studies.

The main strength of the retrospective study is the utilization of the unique Danish registers and databases, allowing us to assess potential drug-drug interactions during the initiation of biological DMARDs. However, only patients treated at one Danish hospital were included. Another strength is the use of a control group, patients treated with adalimumab, a biological drug previously not shown to cause drug-drug interactions [5, 33]. The major limitation of the retrospective study was the use of 4β-hydroxycholesterol to assess the altered activity of CYP3A4. 4β-Hydroxycholesterol concentrations not only depend on CYP3A4 activity but also cholesterol concentrations. Cholesterol concentrations increase within the first months of tocilizumab treatment [34], which could explain the increased 4β-hydroxycholesterol concentrations. However, increased cholesterol levels have also been described during adalimumab treatment [34], but no changes in 4β-hydroxycholesterol nor cholesterol were observed in the adalimumab group. The use of 4β-hydroxycholesterol as a biomarker of CYP3A4 activity has been much debated [35–38], and its utility is yet to be validated.

In conclusion, IL-6 receptor antagonists seem to cause a transient increase in CYP3A4 activity, corresponding to a weak interaction. Among three patients with rheumatoid arthritis, we demonstrated a trend towards increased CYP3A4 activity after 3 weeks of anti-IL-6R therapy, which normalized to baseline after 12 weeks of treatment. Patients with rheumatoid arthritis are potentially at risk of decreased efficacy of drugs with a narrow therapeutic index during the first weeks of anti-IL-6R therapy. Further studies are warranted to fully elucidate the mechanism behind this transient effect.

### Supplementary Information

Below is the link to the electronic supplementary material.

Supplementary file1 (DOCX 190 KB)

## Data Availability

Anonymized data are available from the authors upon reasonable request.
