# Supplementary material for: Cytochrome P450 activity in rheumatoid arthritis patients during continuous IL-6 receptor antagonist therapy
Source: Eur J Clin Pharmacol. 2023 Oct 13;79(12):1687–98. doi: 10.1007/s00228-023-03578-1 (PMC10663184; doi:10.1007/s00228-023-03578-1)
Supplement: Supplementary file 1 — Supplementary file1 (DOCX 190 KB) [file 228_2023_3578_MOESM1_ESM.docx]

Supplementary material

**Novel insight into IL-6 receptor antagonist drug-disease-drug interactions in patients with rheumatoid arthritis**

Ann-Cathrine Dalgård Dunvald^1^ (ORCID: 0000-0001-7574-0909)

Kasper Søltoft^2^ (ORCID: 0000-0002-1111-8050)

Ekta Sheetal^3^

Søren Andreas Just^4^ (ORCID: 0000-0002-3946-5919)

Ida Emilie Brejning Frederiksen^1^

Flemming Nielsen^1^ (ORCID: 0000-0002-5657-405X)

Dorte Aalund Olsen^5^  (ORCID: 0000-0003-2589-738X)

Jonna Skov Madsen^5,6^ (ORCID: 0000-0001-6668-4714)

Oliver Hendricks^7,8^ (ORCID: 0000-0001-9473-5072)

Tore Bjerregaard Stage^1,9^ (ORCID: 0000-0002-4698-4389)

1. Clinical Pharmacology, Pharmacy and Environmental Medicine, Department of Public Health, University of Southern Denmark, Odense, Denmark
2. Department of Rheumatology, Odense University Hospital, Odense, Denmark
3. Department of Rheumatology, Hospital South West Jutland, Esbjerg, Denmark
4. Section of Rheumatology, Department of Medicine, Svendborg Hospital, Odense University Hospital, Svendborg, Denmark
5. Department of Biochemistry and Immunology, Lillebaelt Hospital, Vejle, Denmark
6. Department of Regional Health Research, Faculty of Health Sciences, University of Southern Denmark, Denmark
7. Danish Hospital for Rheumatic Diseases, Hospital South Jutland, Sønderborg, Denmark
8. The DANBIO Registry, Denmark
9. Department of Clinical Pharmacology, Odense University Hospital, Odense, Denmark

**Correspondence**

Tore Bjerregaard Stage

Clinical Pharmacology, Pharmacy and Environmental Medicine

University of Southern Denmark

JB Winsløwsvej 19, 2

DK-5000 Odense C, Denmark

E-mail: tstage@health.sdu.dk

Phone: 0045 65503678

**Figure S1** Individual concentration-time curves for the three patients with rheumatoid arthritis (blue, orange, and purple) before and after three weeks and 12 weeks of IL-6 receptor antagonist therapy.


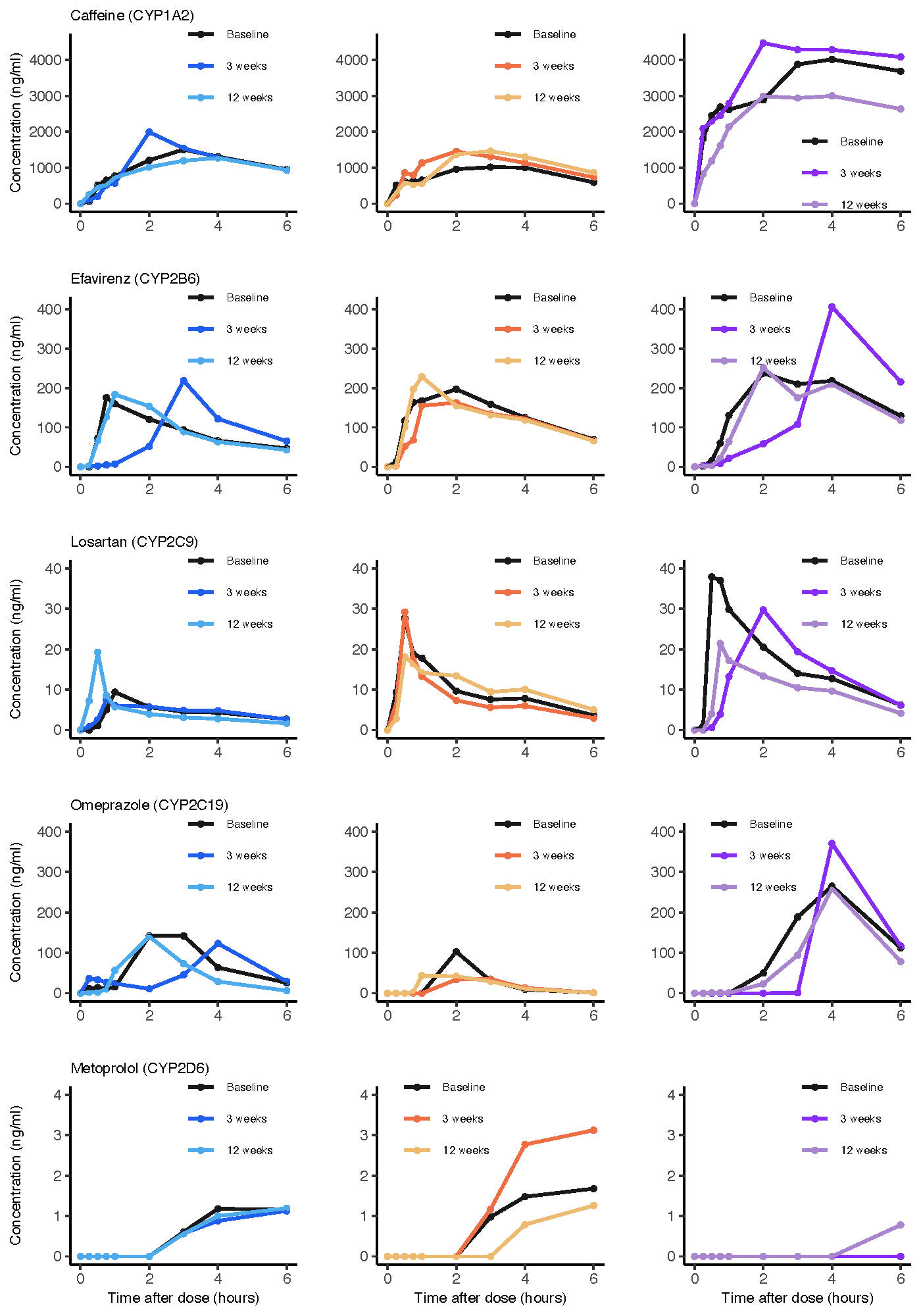


| **Table S1** Pharmacokinetic parameters and metabolic ratios of the probe drugs caffeine (CYP1A2), efavirenz (CYP2B6), losartan (CYP2C9), omeprazole (CYP2C19), and midazolam (CYP3A4). Metoprolol is not shown due to too few data points. Assessed in three patients with rheumatoid arthritis before (baseline) and after 3 weeks and 12 weeks of IL-6 receptor antagonist therapy. | | | | | | |
| --- | --- | --- | --- | --- | --- | --- |
|  | PARAMETER | Baseline  (Median (IQR)) | 3 weeks  (Median (IQR)) | 3 weeks/Baseline  GMR (95% CI) | 12 weeks  (Median (IQR) | 12 weeks/Baseline  GMR (95% CI) |
| CAFFEINE (CYP1A2) | |  |  |  |  |  |
|  | C_max_ (ng ml^-1) | 1504 (1260-2758) | 1989 (1719-3228) | 1.28 (0.93-1.76) | 1459 (1364-2227) | 0.97 (0.41-2.30) |
|  | T_max_ (h) | 3.0 (3.0-3.5) | 2.0 (2.0-2.1) | 0.63 (0.39-1.02) | 4.0 (3.5-4.0) | 1.11 (0.73-1.67) |
|  | Metabolic ratio [caf]/[para]^b^ | 1.56 (1.29-1.84) | 1.55 (1.27-1.74) | 0.96 (0.85-1.08) | 1.61 (1.45-1.73) | 1.05 (0.66-1.66) |
|  | Metabolic ratio [para]/[caf]^b^ | 0.64 (0.56-0.81) | 0.64 (0.58-0.82) | 1.04 (0.93-1.17) | 0.62 (0.58-0.70) | 0.96 (0.60-1.52) |
| EFAVIRENZ (CYP2B6) | |  |  |  |  |  |
|  | C_max_ (ng ml^-1) | 197 (186-217) | 218 (190-312) | 1.21 (0.49-2.99) | 228 (206-240) | 1.09 (0.94-1.25) |
|  | T_max_ (h) | 2.0 (1.4-2.0) | 3.0 (2.5-3.5) | 1.99 (0.36-11.16) | 1.0 (1.0-1.5) | 0.87 (0.25-3.06) |
|  | Metabolic ratio [OH-efa]/[efa]^c^ | 0.17 (0.16-0.28) | 0.19 (0.16-0.29) | 0.99 (0.73-1.32) | 0.23 (0.23-0.32) | 1.27 (0.84-1.92) |
|  | Metabolic ratio [efa]/[OH-efa]^c^ | 5.81 (4.2-6.19) | 5.29 (3.93-6.44) | 1.01 (0.75-1.36) | 4.42 (3.43-4.44) | 0.79 (0.52-1.19) |
| LOSARTAN (CYP2C9) | |  |  |  |  |  |
|  | C_max_ (ng ml^-1) | 27.7 (18.5-32.8) | 29.2 (18.3-29.4) | 0.87 (0.58-1.31) | 19.3 (18.7-20.3) | 0.91 (0.16-5.28) |
|  | T_max_ (h) | 0.5 (0.5-0.8) | 0.8 (0.7-1.4) | 1.47 (0.17-12.86) | 0.5 (0.5-0.6) | 0.91 (0.23-3.61) |
|  | Metabolic ratio [E3174]/[los]^c^ | 11.8 (9.3-12.8) | 14.8 (12.2-17.9) | 1.39 (1.10-1.77) | 13.7 (10.0-15.7) | 1.12 (0.21-5.89) |
|  | Metabolic ratio [los]/[E3174]^c^ | 0.08 (0.08-0.12) | 0.07 (0.06-0.09) | 0.72 (0.57-0.91) | 0.07 (0.06-0.11) | 0.89 (0.17-4.7) |
| OMEPRAZOLE (CYP2C19) | |  |  |  |  |  |
|  | C_max_ (ng ml^-1) | 142 (122-203) | 123 (79-247) | 0.74 (0.12-4.44) | 140 (92-200) | 0.74 (0.23-2.46) |
|  | T_max_ (h) | 2.0 (2.0-3.0) | 4.0 (3.5-4.0) | 1.44 (0.62-3.37) | 2.0 (1.5-3.0) | 0.79 (0.29-2.15) |
|  | Metabolic ratio [OH-ome]/[ome]^b^ | 0.62 (0.47-2.28) | 0.47 (0.36-2.00) | 0.82 (0.67-1.01) | 0.85 (0.59-1.86) | 1.01 (0.46-2.23) |
|  | Metabolic ratio [ome]/[OH-ome]^b^ | 1.62 (0.94-2.40) | 2.13 (1.20-3.00) | 1.21 (0.99-1.49) | 2.13 (1.20-3.00) | 0.99 (0.45-2.17) |
| MIDAZOLAM (CYP3A4) | |  |  |  |  |  |
|  | AUC_0-6h_ (ng*h ml^-1) | 11.2 (8.0-15.0) | 8.8 (6.6-12.0) | 0.84 (0.67-1.04) | 10.7 (7.8-16.8) | 1.07 (0.78-1.48) |
|  | AUC_0-inf_ (ng*h ml^-1) | 13.9 (10.5-19.4) | 11.0 (8.6-14.6) | 0.8 (0.64-0.99) | 13.0 (9.9-21.5) | 1.02 (0.72-1.46) |
|  | C_max_ (ng ml^-1) | 6.44 (5.25-7.86) | 3.94 (2.93-5.09) | 0.58 (0.37-0.91) | 7.13 (5.34-8.81) | 1.03 (0.72-1.47) |
|  | T_max_ (h) | 0.5 (0.5-0.5) | 0.8 (0.7-0.9) | 1.47 (0.61-3.55) | 0.5 (0.5-0.6) | 1.47 (0.61-3.55) |
|  | T_1/2_ (h) | 3.13 (3.10-3.36) | 2.12 (2.02-2.54) | 0.71 (0.34-1.48) | 3.00 (2.65-3.17) | 0.88 (0.60-1.28) |
|  | AUC ratio (AUC mid/AUC OH-mid) | 0.14 (0.10-0.15) | 0.10 (0.08-0.11) | 0.82 (0.54-1.25) | 0.16 (0.11-0.17) | 1.10 (0.82-1.49) |
|  | AUC ratio (AUC OH-mid/AUC mid) | 7.20 (6.81-11.12) | 10.29 (9.14-12.81) | 1.22 (0.80-1.86) | 6.28 (5.97-10.4) | 0.91 (0.67-1.22) |
|  | Metabolic ratio [mid]/[OH-mid]^a^ | 0.12 (0.09-0.13) | 0.08 (0.07-0.09) | 0.82 (0.47-1.44) | 0.15 (0.11-0.15) | 1.16 (0.86-1.57) |
|  | Metabolic ratio [OH-mid]/[mid]^a^ | 8.51 (7.81-12.68) | 12.3 (10.82-14.08) | 1.21 (0.69-2.12) | 6.9 (6.69-10.69) | 0.86 (0.64-1.16) |
|  | CL_f_ (ml h^-1) | 138344 (117032-231732) | 251745 (183853-279014) | 1.28 (0.56-2.91) | 177217 (116328-247446) | 0.90 (0.33-2.45) |
|  | CL_R_ (ml h^-1) | 568 (399-820) | 967 (726-1011) | 1.52 (0.49-4.76) | 837 (775-928) | 1.63 (0.37-7.2) |
| ^a^ Metabolic ratio is assessed at 2 hours. ^b^ Metabolic ratio is assessed at 4 hours. ^c^ Metabolic ratio is assessed at 6 hours.  Abbreviations: AUC: area under the plasma-concentration curve, caf: caffeine, CI: confidence interval, CL_f_: formation clearance of the main metabolite, CL_R_: renal clearance, C_max_: maximum plasma concentration, CYP: cytochrome P450, efa: efavirenz, GMR: geometric mean ratio, h: hours, los: losartan, mid: midazolam, met: metoprolol, OH-efa: 8-hydroxyefavirenz, OH-met: 5-hydroxymetoprolol, OH-mid: α-hydroxymidazolam, OH-ome: hydroxyomeprazole, ome: omeprazole, par: paraxanthine, T_max_: time to maximum plasma concentration | | | | | | |

| **Table S2** 4β-hydroxycholesterol and 4β-hydroxycholesterol/cholesterol ratio as a biomarker for CYP3A4 activity is unchanged in three patients with rheumatoid arthritis before and after 3 weeks and 12 weeks of treatment with an IL-6 receptor antagonist. | | | | | |
| --- | --- | --- | --- | --- | --- |
| PARAMETER | Baseline  Median (IQR) | 3 weeks  Median (IQR) | 3 weeks/Baseline  GMR (95% CI) | 12 weeks  Median (IQR) | 12 weeks/Baseline  GMR (95% CI) |
| Cholesterol 10^-4 ng/mL | 1317 (1273-1,537) | 1532 (1,443-2,722) | 1.42 (0.54-3.75) | 1740 (1,669-2,469) | 1.46 (0.43-4.96) |
| 4β-OHC ng/mL | 19.9 (16.6-26.1) | 26.8 (20.5-27.2) | 1.07 (0.14-8.37) | 16.5 (14.5-24.8) | 0.92 (0.74-1.16) |
| 4β-OHC/Cholesterol ratio *10^4 | 0.16 (0.12-0.21) | 0.09 (0.08-0.15) | 0.75 (0.15-3.77) | 0.10 (0.09-0.1) | 0.63 (0.19-2.13) |
| Abbreviations: 4β-OHC: 4beta-hydroxycholesterol, CI: confidence interval, IQR: interquartile range, GMR: geometric mean ratio. | | | | | |
